# Temporal Dynamics of *Salmonella enterica* subsp. *enterica* Serovar Agona Isolates From a Recurrent Multistate Outbreak

**DOI:** 10.3389/fmicb.2020.00478

**Published:** 2020-03-23

**Authors:** Maria Hoffmann, John Miller, David Melka, Marc W. Allard, Eric W. Brown, James B. Pettengill

**Affiliations:** ^1^Division of Microbiology, Office of Regulatory Science, Center for Food Safety and Applied Nutrition, U.S. Food & Drug Administration, College Park, MD, United States; ^2^Division of Public Health and Biostatistics, Office of Food Defense, Communication and Emergency Response, Center for Food Safety and Applied Nutrition, U.S. Food & Drug Administration, College Park, MD, United States

**Keywords:** dormancy, outbreak, *Salmonella*, whole genome sequencing, PacBio, SNP analyses

## Abstract

The largest outbreak of *Salmonella* Agona in the United States occurred in 1998. It affected more than 400 patients and was linked to toasted oat cereal. Ten years later, a similar outbreak occurred with the same outbreak strain linked to the same production facility. In this study, whole-genome sequence (WGS) data from a set of 46 *Salmonella* Agona including five isolates associated with the 1998 outbreak and 25 isolates associated with the 2008 outbreak were analyzed. From each outbreak one isolate was sequenced on the Pacific Biosciences *RS II* Sequencer to determine the complete genome sequence. We reconstructed a phylogenetic hypothesis of the samples using a reference-based method for identifying variable sites. Using Single Nucleotide Polymorphism (SNP) analyses, we were able to distinguish and separate *Salmonella* Agona isolates from both outbreaks with only a mean of eight SNP differences between them. The phylogeny illustrates that the 2008 outbreak involves direct descendants from the 1998 outbreak rather than a second independent contamination event. Based on these results, there is evidence supporting the persistence of *Salmonella* over time in food processing facilities and highlights the need for consistent monitoring and control of organisms in the supply chain to minimize the risk of successive outbreaks.

## Introduction

*Salmonella enterica* subsp. *enterica* is comprised of more than 1,500 serovars, including *Salmonella enterica* subsp. *enterica* serovar Agona (*Salmonella* Agona). *Salmonella* Agona was first isolated from cattle in Ghana in 1952 (Guinee et al., 1964). Within the last several years, *Salmonella* Agona has been one of the top 20 most commonly reported serotypes causing human infections (CDC, 2014). It has been responsible for numerous human outbreaks associated with various dry food products such as dried milk, machacado, dried snacks, and cereal (Clark et al., 1973; Killalea et al., 1996; Taylor et al., 1998). In the United States, the largest known outbreak of *Salmonella* Agona infections occurred between April and May in 1998 (Russo et al., 2013). During that time, the Centers for Disease Control and Prevention (CDC) investigated a multistate (11 states) outbreak of *Salmonella* Agona infections comprised of 209 confirmed cases (Centers for Disease Control and Prevention, 1998). Among the 162 case patients for which there was available information, 46 (28%) had been hospitalized (Centers for Disease Control and Prevention, 1998). Collaborative investigative efforts by state and federal officials implicated toasted oat cereal as the source of this Agona outbreak. The pulsed-field gel electrophoresis (PFGE) *Xba*l pattern (JABX01.0001) of the Agona isolate from the cereal production facility was indistinguishable from the predominant PFGE pattern among outbreak-associated clinical isolates. After the investigation, the implicated production lines from the company’s plant were sealed off, all equipment was removed, all surfaces were stripped to bare concrete, decontaminated, refinished and new production lines were installed (Russo et al., 2013).

A decade later, in April 2008, the CDC announced a 15-state outbreak associated with *Salmonella* Agona resulting in 28 identified cases, with eight individuals being hospitalized. The federal investigation suggested that unsweetened puffed-rice cereals and unsweetened puffed-wheat cereals from the same company that was associated with the 1998 outbreak were the likely sources of contamination (CDC, 2008). The PFGE pattern of the 2008 outbreak strain had the same *Xba*l pattern (JABX01.0001) as of *Salmonella* Agona identified during the 1998 outbreak associated with toasted oat cereal by company X. On April 5, 2008 the same company X voluntarily recalled its unsweetened puffed-rice cereals and unsweetened puffed-wheat cereals after finding *Salmonella* Agona contamination during routine testing from the same plant (Russo et al., 2013).

In this study, whole genome sequence (WGS) data from a group of closely related *Salmonella* Agona isolates were analyzed. The majority of the isolates were investigated as a part of the two outbreaks of salmonellosis associated with cereals that were separated by 10 years and our objective was to determine the evolutionary relationships among these outbreak isolates as a means to differentiate among two competing hypotheses: (1) persistence of *Salmonella* within the facility and (2) reintroduction of the same strain. We also demonstrate how the use of WGS can identify stable SNP targets that can be utilized for differentiating closely related *Salmonella* Agona isolates and clustering closely related isolates together, such as those of the 1998 and 2008 outbreak. Further, we show that the 2008 outbreak involves direct descendants from the 1998 outbreak rather than a second independent event.

## Materials and Methods

### Bacterial Isolates

We analyzed 46 *Salmonella* Agona whole genome sequences, 31 of which are first reported here while the remainder were publicly available from GenBank at NCBI (Table 1). Of these 46 isolates, 30 were linked with the original investigation into the outbreak of salmonellosis associated with breakfast cereal including one clinical isolate and 4 food isolates obtained in 1998 and 5 clinical isolates, 8 food isolates, and 12 environmental isolates acquired in 2008. All 30 outbreak associated isolates exhibited indistinguishable *Xba*l PFGE patterns (JABX01.0001). We also included one clinical *Salmonella* Agona isolate SL483 containing the same *Xba*l PFGE pattern from a different event isolated in Wisconsin 2003. The remaining *Salmonella* Agona isolates were chosen to provide a measure of the genetic diversity that is found among other isolates within the serovar.

**TABLE 1:** Isolates information for the *Salmonella* Agona included in this study.

**Strain name**	**Year**	**Country**	**Host**	**PFGE profile *Xba*I**	**PFGE profile *Bln*I**	**Platform**	**Genomesize (Mb)**	**WGS accession/SRA #**
**Isolates sequenced in this study**								
CFSAN000449	1999	United States	Cattle	N/A	N/A	454 FLX	4.9	AYDT00000000
CFSAN000451	2005	United States	Soybean meal	JABX01.0050	N/A	454 FLX	4.7	AYCL00000000
CFSAN000452	2008	United States	Environmental	JABX01.0001	JABA26.0339	454 FLX	4.7	AYDF00000000
CFSAN000453	2008	United States	Environmental	JABX01.0001	JABA26.0001	454 FLX	4.7	AYDE00000000
CFSAN000454	2008	United States	Environmental	JABX01.0001	JABA26.0001	454 FLX	4.7	AYDD00000000
CFSAN000455	2008	United States	Environmental	JABX01.0001	JABA26.0001	454 FLX	4.7	AYDC00000000
CFSAN000456	2008	United States	Environmental	JABX01.0001	JABA26.0338	454 FLX	4.7	AYDB00000000
CFSAN000457	2008	United States	Environmental	JABX01.0001	JABA26.0001	454 FLX	4.7	MAOW00000000
CFSAN000458	2008	United States	Environmental	JABX01.0001	JABA26.0001	454 FLX	4.6	LXTN00000000
CFSAN000459	2008	United States	Environmental	JABX01.0001	JABA26.0001	454 FLX	4.7	LYVG00000000
CFSAN000460	2008	United States	Environmental	JABX01.0001	JABA26.0001	454 FLX	4.7	LXTO00000000
CFSAN000461	2008	United States	Environmental	JABX01.0001	JABA26.0001	454 FLX	4.7	MAOV00000000
CFSAN000462	2008	United States	Environmental	JABX01.0001	JABA26.0001	454 FLX	4.7	MAOU00000000
CFSAN000463	2008	United States	Environmental	JABX01.0001	JABA26.0341	454 FLX	4.7	MAOT00000000
CFSAN000470	2008	United States	Cereal	JABX01.0001	JABA26.0001	454 FLX	4.7	AYDH00000000
CFSAN000471	1998	United States	Cereal	JABX01.0001	JABA26.0013	Pacbio	4.7	CP015024
CFSAN000472	2008	United States	Puffed wheat cereal	JABX01.0001	JABA26.0001	454 FLX	4.7	AYCS00000000
CFSAN000473	2008	United States	Puffed wheat cereal	JABX01.0001	JABA26.0001	454 FLX	4.7	AYCR00000000
CFSAN000474	2008	United States	Puffed rice cereal	JABX01.0001	JABA26.0001	454 FLX	4.7	AYCQ00000000
CFSAN000475	2008	United States	Puffed rice cereal	JABX01.0001	JABA26.0001	454 FLX	4.7	AYCP00000000
CFSAN000476	2008	United States	Puffed rice cereal	JABX01.0001	JABA26.0340	454 FLX	4.7	AYCO00000000
CFSAN000477	2008	United States	Puffed rice cereal	JABX01.0001	JABA26.0001	Pacbio	4.7	CP011259
CFSAN000481	2008	China	Mussels	JABX01.0556	N/A	454 FLX	4.8	AYCW00000000
CFSAN000482	2009	China	Baby clams	no pulsenet # associated	N/A	454 FLX	5.0	AYCV00000000
CFSAN000486	2007	Mexico	Herbal tea	JABX01.0225	N/A	454 FLX	4.8	AOZV00000000
CFSAN000501	2008	United States	Clinical	JABX01.0001	JABA26.0001	454 FLX	4.7	AYDV00000000
CFSAN000502	2008	United States	Clinical	JABX01.0001	JABA26.0001	454 FLX	4.7	AYDW00000000
CFSAN000503	2008	United States	Clinical	JABX01.0001	JABA26.0035	454 FLX	4.7	AYDX00000000
CFSAN000504	1998	United States	Cereal	JABX01.0001	JABA26.0001	454 FLX	4.7	AYDK00000000
CFSAN000505	1998	United States	Cereal	JABX01.0001	JABA26.0013	454 FLX	4.7	AYDL00000000
CFSAN003041	2008	United States	Puffed rice	JABX01.0001	JABA26.0001	Miseq	4.7	SRR949597
**Isolates for which the sequence was obtained from NCBI**								
ERR178928	2005	Ireland	Environment	AgoX63	N/A	Hiseq 2000	4.8	CARE01000001
ERR178921	1987	Austria	Clinical	AgoX76	N/A	Hiseq 2000	4.8	CARL01000001
ERR178919	2008	Scotland	Clinical	SAGOXB.0066	N/A	Hiseq 2000	4.8	CARN01000001
ERR178910	2008	Ireland	Pork	SAGOXB.0066	N/A	Hiseq 2000	4.7	CARV01000001
ERR178902	2009	Ireland	Environment	AgoX67	N/A	Hiseq 2000	4.8	CASB01000001
ERR178895	2002	Germany	Aniseed	SAGOXB.0010	N/A	Hiseq 2000	4.8	CASH01000001
ERR178894	2000	Scotland	Clinical	SAGOXB.0020	N/A	Hiseq 2000	4.8	CASI01000001
ERR178887	2008	United States	Clinical	JABX01.0001	JABA26.0001	Hiseq 2000	4.8	CASO01000001
ERR178886	2008	United States	Clinical	JABX01.0001	JABA26.0001	Hiseq 2000	4.7	CASP01000001
ERR178885	1998	United States	Clinical	JABX01.0001	JABA26.0001	Hiseq 2000	4.8	CASQ01000001
ERR178878	2003	Germany	Fennel	SAGOXB.0002b	N/A	Hiseq 2000	4.8	CASW01000001
ERR178871	2006	Ireland	Poultry	SAGOXB.0066	N/A	Hiseq 2000	4.7	CATD01000001
ERR178861	2005	Ireland	Clinical	AgoX3	N/A	Hiseq 2000	4.9	CATP01000001
ERR178884	1998	United States	Toasted oats cereal	JABX01.0001	JABA26.0001	Hiseq 2000	4.8	CATX01000001
SL 483	2003	United States/WI	Clinical	JABX01.0001	JABA26.0005	3730xl DNA analyzer	4.8	CP001138

### DNA Preparation, PFGE, and Genome Sequencing

Pulsed-field gel electrophoresis was performed according to the PulseNet protocol of the Center for Disease Control and Prevention^1^ (CDC). Genomic DNA of each strain was isolated from overnight cultures using DNeasy Blood and Tissue kit (Qiagen, CA, United States). Twenty-eight isolates were shotgun sequenced with the Roche Genome Sequencer FLX (454 Life Sciences, Roche, Branford, CT, United States) using the GS FLX Titanium Sequencing Kit XLR70 according to the manufacturer’s protocol. One isolate was sequenced using Illumina’s MiSeq platform (Illumina, Inc., CA, United States). Sample preparation and the sequencing library was prepared using the Nextera XT Sample Preparation Kit and then sequenced for 2 × 151 cycles. Two outbreak associated isolates (1998 and 2008) were sequenced on the Pacific Biosciences (PacBio) *RS* II Sequencer and assembled as previously described (Hoffmann et al., 2015). Specifically, a single SMRTbell 10-kb library was prepared according to the 10-kb PacBio sample preparation protocol and sequenced using C2 chemistry on two single-molecule real-time (SMRT) cells with a 180-min collection protocol. The 10-kb continuous long read (CLR) data were *de novo* assembled using the PacBio hierarchical genome assembly process (HGAP3)/Quiver software package, followed by Minimus2, and polished by Quiver (Chin et al., 2013). The assembled sequences were annotated using the NCBI Prokaryotic Genomes Annotation Pipeline (PGAP) and have been deposited at DDBJ/EMBL/GenBank.

### Construction of SNP Matrices and Phylogenetic Inference

We used the CFSAN SNP Pipeline^2^ (Davis et al., 2015) to construct a SNP matrix among the 46 individuals. This pipeline is a reference-based approach within which Bowtie2 (Langmead and Salzberg, 2012) is used to align reads to the reference and VarScan (Koboldt et al., 2012) is used to detect variants. Within the pipeline, we used *Salmonella* Agona SL483 (CP001138) as the reference; using NCBI’s methodology (Ciufo et al., 2018) CP001138 has a type strain ANI of 98.6994 confirming taxonomic accuracy. We applied a SNP density filter (i.e., three or more SNPs in a 1000 bp window were removed) to remove variants that are likely the result of recombination or mobile elements (e.g., Petkau et al., 2017).

With the resulting SNP matrix from the CFSAN SNP Pipeline, we constructed a phylogenetic hypothesis of the evolutionary relationships among the 46 isolates investigated using the Genetic Algorithm for Likelihood Inference (GARLI; v2.01.1067; Zwickl, 2006). We used the default settings within GARLI and the best tree was chosen among 10 replicate runs using the observed SNP matrix; topological support was estimated by conducting 1,000 bootstrap replicates. Trees were rooted based on preliminary analyses including the serovar Soerenga as an outgroup. We also applied the ACCTRAN method within the R package phangorn (Schliep, 2011) to provide the number of changes along branches of the best tree from GARLI. We used PopART to construct a minimum spanning network (Bandelt et al., 1999) using the SNP matrix.

### Functional and Genomic Differences

A custom python script was written to determine the position of each SNP in the reference genome, the gene within which it was found, and the type of mutation (synonymous or non-synonymous). A custom script was also used to identify fixed SNPs between the outbreak clade and all other isolates. Both scripts are available at https://github.com/CFSAN-Biostatistics/snps_in_genes. Using the COG information in the feature table for the reference found in the Genome database at NCBI, we then assigned each gene to one of the higher functional categories. We compared the abundance of each category between the isolates found in the outbreak and the complete dataset, which indicates what functions might be more pronounced in the former.

To further explore genomic differences among the isolates, the complete chromosomes were aligned with MAUVE aligner version 20150226 using progressive algorithm with default settings (Darling et al., 2004). We constructed a circle plot of genomic differences between the publicly available Agona strain SL483 and the two completely closed outbreak associated isolates CFSAN000477 and CFSAN000471using BRIG (Alikhan et al., 2011).

## Results and Discussion

Public health authorities were informed in 1998 and 2008 of outbreaks involving *Salmonella* Agona that resulted from consumption of contaminated cereal from the same company X. The PFGE pattern of these highly clonal Agona isolates from 1998 and 2008 were indistinguishable. At that time PFGE was the method used for traceback during an outbreak investigation. However, PFGE does not have the resolution to distinguish a transient strain from a resident strain (Pightling et al., 2018) and, therefore, at that time using this method officials from the state health department were able to correctly identify the source of an outbreak but were not able to determine if the 2008 Agona isolate is the same strain as the 1998 Agona isolate.

Since 2012, several studies have demonstrated the higher resolution and increased discriminatory power of WGS relative to that of PFGE where the former can differentiate highly clonal isolates. This increased resolution of WGS is critical in a successful investigation of foodborne outbreaks to determine the source(s) of contamination (Hoffmann et al., 2014, 2016; Allard et al., 2018). Thus, WGS was invoked retrospectively to the cereal outbreaks of 1998 and 2008 in an attempt to resolve and ameliorate the shortcomings of the other more traditional molecular strategies used for such purposes, as well as to characterize the dynamics of the two outbreak scenarios.

**FIGURE 1:**
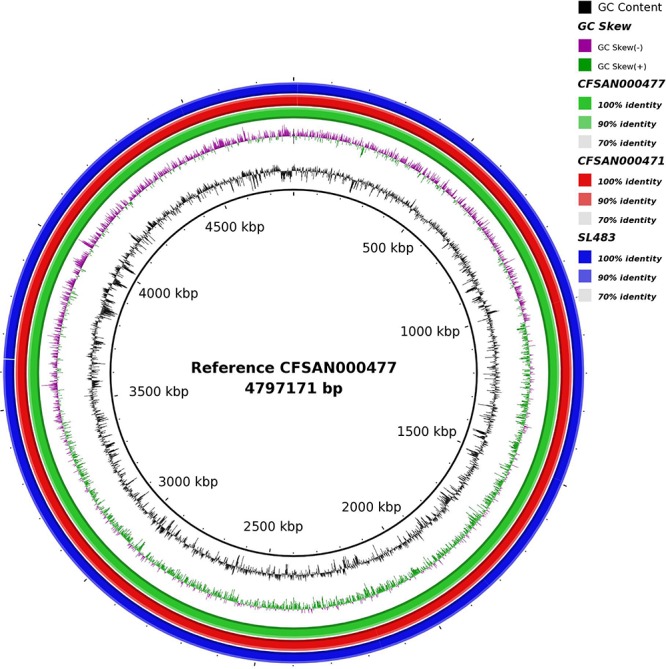
Circle plot showing the similarity between three closed *Salmonella* Agona isolates associated with the cereal outbreaks. The reference *Salmonella* Agona CFSAN000477 is associated with the 2008 outbreak, *Salmonella* Agona CFSAN000471 is associated with the 1998 outbreak and *Salmonella* Agona SL483 is associated with a sporadic case in 2003.

First, using long read sequencing the complete genome sequences of *Salmonella* Agona isolate CFSAN000471 obtained from cereal during the outbreak in 1998 and of *Salmonella* Agona isolate CFSAN000477 obtained from unsweetened puffed-rice cereal during the outbreak in 2008 (Hoffmann et al., 2015) were sequenced with the Single Molecule, Real-Time (SMRT) sequencing technology. Isolate CFSAN000471 was sequenced with 110× coverage; its genome size was 4,798,531 bp, and the G + C content was 52.1% and Isolate CFSAN000477 was sequenced with 130× coverage; its genome size was 4,797,172 bp, and the G + C content was also 52.1%. At the time *Salmonella* Agona SL483 was the only complete genome from *Salmonella* Agona available in Genbank; it had a genome size of 4,798,660 bp, and a G + C content of 52.1%. The strain was isolated from a clinical sample and is associated with a sporadic case in 2003. Figure 1 shows the pronounced similarity between these three closed *Salmonella* Agona isolates suggesting that they are highly related with each other. To further characterize them we used the MAUVE aligner to align them and checked manually for differences. The different genome sizes can be explained by the insertion/deletion of transposases. CFSAN000477 and CFSAN000471 carry the same transposase but at different locations within the chromosome (CFSAN000471 transposase insertion: 710,112–711,499; CFSAN000477 (transposase insertion: 987,888–989,229) while SL483 is missing the specific transposase but instead carries an insertion sequence element, IS10, at three different loci. Further, very few SNPS and deletions and insertions were found among the three isolates listed in Supplementary Table 1. Explicitly, CFSAN000471 isolated in 1998 showed only one unique informative SNP in gene *int* (locus_tag = SEEA8692_015865) not found in CFSAN000477 and SL483. SL483 has one unique informative SNP in gene *hslO* (locus_tag = SeAg_B3697), two unique SNPs, two unique deletions and two unique insertions in non-coding region, and is missing a hypothetical protein. CFSAN000477 has six unique including five informative SNPs [*fusA* (locus_tag = SEEA0421_002795), GDP-mannose pyrophosphatase (locus_tag = SEEA0421_021175), transporter gene (locus_tag = SEEA0421_019215), protease (locus_tag = SEEA0421_011315), hypothetical protein (locus_tag = SEEA0421_010320), and fructose-6-phospharte aldolase A (locus_tag = SEEA0421_006170)], four SNPs, one insertion and one deletion in non-coding region.

**FIGURE 2:**
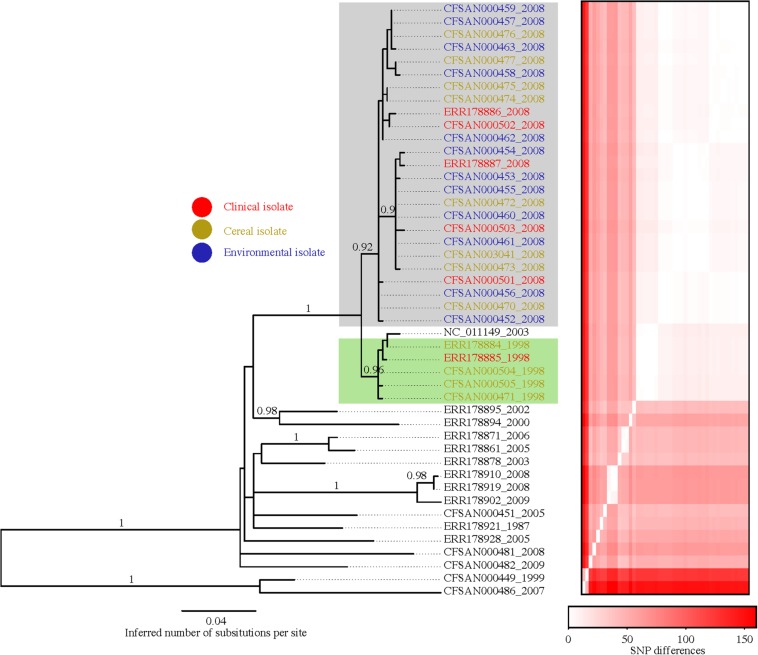
Phylogenetic reconstruction of the 46 *Salmonella* Agona isolates investigated herein. The tree was constructed from a SNP matrix obtained with the CFSAN SNP Pipeline within which the isolate *Salmonella* Agona SL483 was used as the reference. The program GARLI v2.01.1067 was used to infer the tree. The heatmap to the right is ordered according to the tips of the tree and shows the pairwise SNP distances among isolates (e.g., the first two columns and last two rows represent the two most basal isolates in the tree that are very different from all other isolates but similar to one another).

The genomic size (including extrab. -chromosomal DNA) of the 46 *Salmonella* Agona isolates varied from 4.6 to 5.1 Mb. Primarily, differences in genomic size was due to the presence or absence of mobile genetic elements, such as phages and genetic islands. Using the complete genome of *Salmonella* Agona (SL483), we were able to more accurately map the raw reads to the reference genome to build a SNP matrix and subsequently construct a phylogenetic tree (Figure 2). Overall, we identified 441 variable SNPs with 112 not located on a gene. Supplementary Table 2 lists all 441 SNPs including SNP position in the genome, description of the gene where the SNP is located, SNP position in the gene, amino acid change and the substitution type; Supplementary Figure S1 shows the number of changes along branches of the phylogenetic tree inferred with GARLI.

The data derived from the phylogenetic analysis of 46 *Salmonella* Agona isolates (Table 1), collected from various sources between 1987 and 2009 including 5 isolates associated with the 1998 outbreak, and 25 isolates associated with the 2008 outbreak provided critically relevant information. First and most importantly, the 1998 and 2008 outbreak associated isolates clustered together to the exclusion of other isolates with strong support (i.e., 19 fixed SNPs and 100% bootstrap support). Second, the tree showed that all of the isolates associated with the 2008 outbreak, including 6 clinical, 7 cereal, and 12 environmental isolates from the implicated food production facility clustered together with 92% bootstrap support (Figure 2) and three fixed SNPs. Third, the two clinical and three food isolates associated with the 1998 outbreak clustered together with 96% bootstrap support and four fixed SNPs. Interesting, the reference isolate SL483 grouped together with the 1998 outbreak isolates. SL483 is a clinical isolate (sporadic case) obtained in 2003. The analyses suggest that most likely the patient was infected with the same *Salmonella* Agona strain. Interestingly, there are also two strains from 1998 and two strains from 2008 that were part of a previous publication Zhou et al., 2013 that also cluster with the sample associated with the particular cereal outbreak. The metadata for those samples indicates that they indeed are part of the outbreak event [“A food isolate, isolated at the time of the (1998| 2008) outbreak in the United States”]. Fourth, there is a tendency for isolates to cluster based on geographical location such as United States, Europe and China and proximity. The most distinct isolate in this study was collected from herbal tea in Mexico in 2007.

**FIGURE 3:**
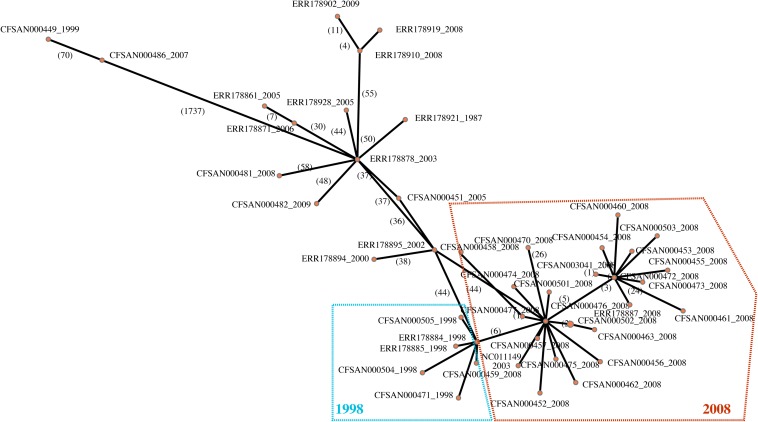
A minimum spanning network constructed with popArt (http://popart.otago.ac.nz/) showing the genetic similarity among 46 *Salmonella* Agona isolates. Input for the network was the SNP matrix constructed with the CFSAN SNP Pipeline. Edge labels represent the number of SNP differences between nodes.

**FIGURE 4:**
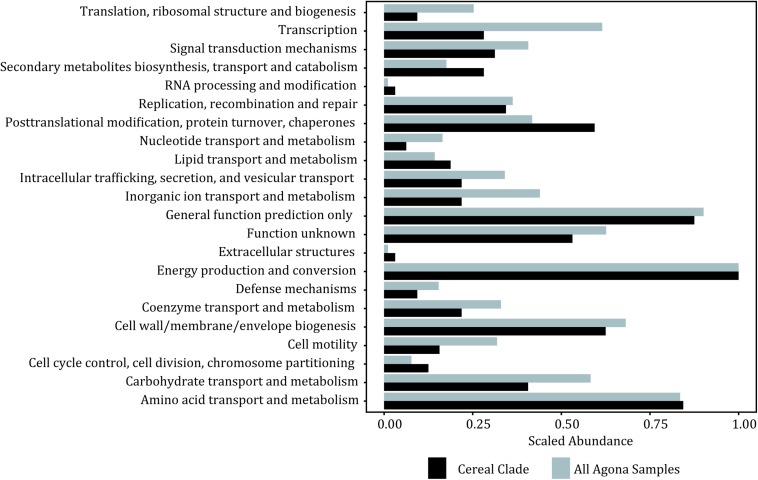
Histogram showing the counts of mutations found within each protein type (COG category) between the outbreak clade and all samples.

Given the importance of the tree topology to the accurate interpretation of this study results, we constructed using the same SNP matrix a minimum spanning tree (Figure 3) to further illustrate the relationships among the isolates and to provide additional insight into whether the observed phylogenetic relationships may be influenced by recombination and/or mobile elements. With the phylogenetic analyses linking the 1998 and 2008 strains, the network analysis illustrates that the outbreak isolates form a star-like clade (Figure 3). Star-like clusters are often attributed to rapid diversification and/or a clonal swarm. In this situation, it reveals additional evidence that indeed the 2008 outbreak are direct descendants from the 1998 outbreak rather than a second independent contamination event. Therefore, our analyses suggest that company X had a resident strain surviving inside their facility for a minimum of a decade. Given that the facility was thought to have taken appropriate measures to eliminate the presence of *Salmonella*, the question arose as to how a strain found 10 years earlier could have persisted in the plant and caused the subsequent outbreak in 2008. One possible explanation is that the bacteria persisted in the cement walls and there is evidence for *Salmonella* forming dense biofilms on such a surface (Joseph et al., 2001). Based on our SNP analyses where variable positions were mapped to an annotated reference genome, there does appear to be fewer number of SNP differences within genes linked to cell mobility, intracellular transport and transcription within the cereal clade (Figure 4). Such evolutionary changes may be driven by selection pressures from bacterial controls instituted by the food industry or by production processes, resulting in enhanced survival, persistence and even growth within food matrices and in the production environment. These increase the likelihood of foodborne outbreaks with morbidity and mortality that threaten public health. To prevent such outbreaks, we must identify the genetic factors responsible for pathogen persistence across the farm to fork supply chain.

## Conclusion

The subtyping of isolates associated with a foodborne outbreak event is essential for successful investigation and eventual traceback to a specific food or environmental source (Allard et al., 2012). In this regard, PFGE has been thought of as the “gold standard” by augmenting public health investigations with information regarding genetic similarity of isolation for nearly two decades (Bergholz et al., 2014). However, numerous clonal strains, a particularly common phenomenon within *Salmonella*, confound epidemiological investigations because PFGE and other traditional molecular typing tools cannot separate these clonally related strains (CDC, 2010). The population and evolutionary dynamics associated with clonal bacterial populations can now be investigated with much greater resolution afforded by next generation sequencing (NGS) data. We are now at a stage that WGS can identify if a company’s product is contaminated with a new strain of a pathogen from the environment or if they have a resident strain within their facility that has persisted over a period of time (e.g., multiple years).

In this study, WGS data from a set of *Salmonella* Agona isolates were analyzed to provide insight into the evolutionary relationships among strains linked to two outbreaks of salmonellosis separated by ten years. All of the *Salmonella* Agona strains associated with the 1998 and 2008 outbreaks could be traced to the same cereal facility in the United States and the analyses of genomic data suggests that the same strain from 1998 was responsible for the 2008 outbreak and it was able to survive in the facility. This highlights the persistence of *Salmonella* and that it can survive in dry food production environments for years.

## Data Availability Statement

GenBank accession numbers for all new sequences are listed in Table 1.

## Author Contributions

All authors played an integral part of project conception. Each author has read and approved the final version of the manuscript. Specifically, MH, MA, EB, and JP conceived and designed the experiments. MH performed the experiments. JP, MH, JM, and DM analyzed the data. MH and JP wrote the manuscript.

## Conflict of Interest

The authors declare that the research was conducted in the absence of any commercial or financial relationships that could be construed as a potential conflict of interest.

## Footnotes

1 http://www.cdc.gov/pulsenet/pathogens/index.html

2 https://github.com/CFSAN-Biostatistics/snp-pipeline
