# Supplementary figures and images for: Temporal Dynamics of Salmonella enterica subsp. enterica Serovar Agona Isolates From a Recurrent Multistate Outbreak
Source: Front Microbiol. 2020 Mar 23;11:478. doi: 10.3389/fmicb.2020.00478 (PMC7104706; doi:10.3389/fmicb.2020.00478)

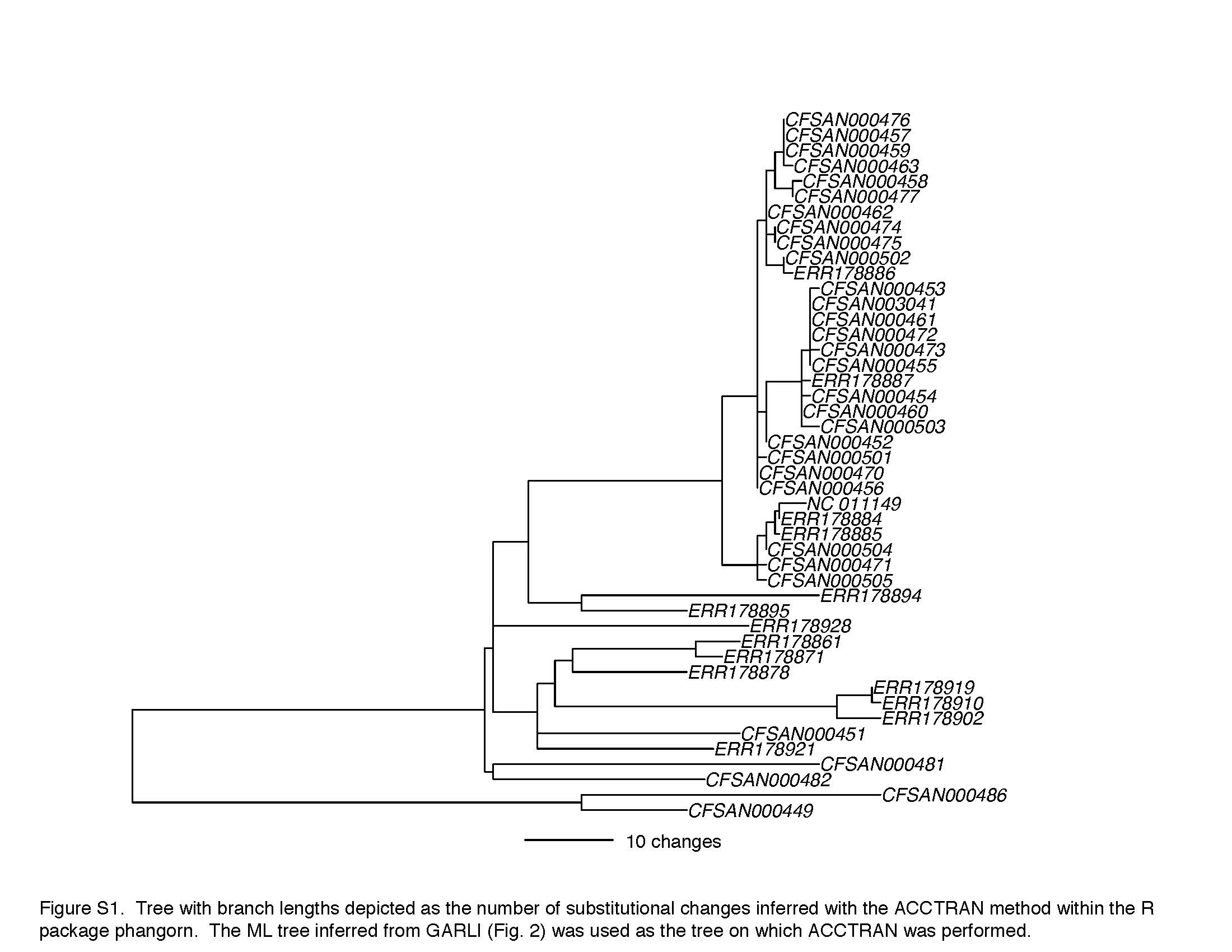

Supplement: Supplementary file 1 [file Image_1.JPEG]
